# The effects of loneliness and isolation on depressive disorder: a narrative review

**DOI:** 10.1192/j.eurpsy.2023.1763

**Published:** 2023-07-19

**Authors:** H. T. J. Cooper, S. De Souza

**Affiliations:** University of Bristol, Bristol, United Kingdom

## Abstract

**Introduction:**

The recent global pandemic has led, for many, to a period of enforced isolation. Anecdotally we are now seeing a “fourth wave” of morbidity. This is not a further wave of Covid infections but a surge of people presenting with depression. Given the potential importance of loneliness and isolation as risk factors for depressive symptoms, we sought to review the literature on this topic.

**Objectives:**

What is the impact of loneliness and isolation on the development and perpetuation of depression?

**Methods:**

A search of the literature was carried out using Medline via OvidSP and Web of Knowledge core collection. Search terms used on Web of Science were ‘lonel*’ AND ‘depress*’, ‘isolat*’ AND ‘depress*’. OvidSP utilised search terms ‘lonel*’ AND ‘depression’, ‘isolation’ AND ‘depression’. MeSH terms were incorporated into the OvidSP search: these included ‘loneliness’, ‘social isolation’, ‘depression’, ‘depression, postpartum’. Papers were filtered first by publication year and article type, then manually through the review of titles and objectives. Full texts of relevant papers were obtained, reviewed and appraised to see if they could help to answer the study question.

**Results:**

Several key themes emerged across the papers reviewed.

Loneliness may be a risk factor for the development of depression and vice versa (Van As *et al*. International Psychogeriatrics 2022; 34(7) 657–69).

Equally, they may have a common aetiology for example shared genetic factors (Achterbergh *et al.* BMC Psychiatry 2020; 20(1) 1-23).

The experience of loneliness was shown to create a cyclical pattern in which feelings of loneliness worsen the deterioration of depression, which in turn worsens the proceeding loneliness. (Wahid *et al.* Child and Adolescent Psychiatry and Mental Health 2022; 16(1), 1-17). Loneliness has also been shown to increase the likelihood of reoccurrence of depression, as well as negatively predicting for recovery from depression (Gabarrell-Pascuet *et al.* Depression and anxiety 2022; 39(2) 147-155, Van As *et al*. International Psychogeriatrics 2022; 34(7) 657–69).

Fear of disclosing depressive symptoms to friends, due to fear of rejection and subsequent social isolation resulting in loneliness, was shown to lead individuals to withdraw from their relationships. This brought about social isolation: consequences of this isolation, such as loss of meaningful relationships, are in themselves risk factors for depression (Caputi *et al.* The Journal of Genetic Psychology 2017; 178(4) 207-216).

**Conclusions:**

To prevent and improve recovery from depression, it is important to consider the importance of loneliness and isolation as risk factors. Consideration should be given to treating both using a biopsychosocial approach.

**Disclosure of Interest:**

None Declared

